# Parameter Estimation for Two-Dimensional Incoherently Distributed Source with Double Cross Arrays

**DOI:** 10.3390/s20164562

**Published:** 2020-08-14

**Authors:** Tao Wu, Yiwen Li, Zhenghong Deng, Bo Feng, Xinping Ma

**Affiliations:** 1 Equipment Management and UAV College, Air Force Engineering University, Xi’an 710051, China; fengbo876@163.com; 2 Aeronautical Engineering College, Air Force Engineering University, Xi’an 710051, China; lee_yiwen@nwpu.edu.cn; 3 Science and Technology on Combustion, Thermal-Structure and Internal Flow Laboratory, Northwestern Polytechnical University, Xi’an 710051, China; 4 School of Automation, Northwestern Polytechnical University, Xi’an 710072, China; dthree@nwpu.edu.cn; 5 College of Resources, Environment and History and Culture, Xianyang Normal University, Xianyang 712000, China; maxinping_2007@126.com

**Keywords:** incoherently distributed sources, direction of arrival, angular spreads, generalized manifold matrix, double cross arrays

## Abstract

A direction of arrival (DOA) estimator for two-dimensional (2D) incoherently distributed (ID) sources is presented under proposed double cross arrays, satisfying both the small interval of parallel linear arrays and the aperture equalization in the elevation and azimuth dimensions. First, by virtue of a first-order Taylor expansion for array manifold vectors of parallel linear arrays, the received signal of arrays can be reconstructed by the products of generalized manifold matrices and extended signal vectors. Then, the rotating invariant relations concerning the nominal elevation and azimuth are derived. According to the rotating invariant relationships, the rotating operators are obtained through the subspace of the covariance matrix of the received vectors. Last, the angle matching approach and angular spreads are explored based on the Capon principle. The proposed method for estimating the DOA of 2D ID sources does not require a spectral search and prior knowledge of the angular power density function. The proposed DOA estimation has a significant advantage in terms of computational cost. Investigating the influence of experimental conditions and angular spreads on estimation, numerical simulations are carried out to validate the effectiveness of the proposed method. The experimental results show that the algorithm proposed in this paper has advantages in terms of estimation accuracy, with a similar number of sensors and the same experimental conditions when compared with existing methods, and that it shows a robustness in cases of model mismatch.

## 1. Introduction

In view of the problem of localization, traditional parameter estimation algorithms are based on a point source model. When spatial scattering characteristics of targets can be ignored and propagations are supposed to be a single straight path between targets and receive arrays, the point target model can simplify the calculation. However, in practical applications, due to scattering, reflection, diffraction and refraction in a complex environment, a large number of multipath phenomena result in the signal source expanding at a certain angle in space, which often has more complex spatial distribution characteristics than the point signal source, and distributed source models are presented in this context [1]. The distribution source exists in two situations. First, the size of the target cannot be ignored when compared with the distance. At this time, the reflection intensity of different parts of the target is random, and the direction is also variable. For instance, in an underwater sonar background, with the reduction of distance, the spatial geometry of a target cannot be ignored, many parts of the target reflecting signals; the point target model cannot describe the characteristics of the source effectively, and distributed sources models are suitable in this context. Secondly, due to the complex environment near the target, local strong scattering occurs. For example, with wireless communications in an environment with high-rise buildings, the intensity of the reflected signal changes with the orientation [2]. There are also similar scenarios, such as the area target or clutter involved in radar signal processing, whose array observation signal usually has a certain diffusion in the spatial and temporal domain [3,4]. At this time, the parameters estimation of targets based on a point source model assumption is seriously deteriorated, while the algorithms based on a distributed source model can obtain more accurate information.

The distributed signal source model has a broad application prospect in radar, sonar and mobile communication because it adopts the signal model, which is more in line with the actual signal characteristics. A distributed source can be thought of as an aggregation of point sources, which are also called scatterers, in a spatial range with a certain density distribution. According to the dependence of scatterers on the time domain, sources are divided into incoherently distributed (ID) sources and coherently distributed (CD) sources. The spatial distribution characteristics of ID and CD sources are described by the angular power density function (ADPF) and angular signal distribution function (ASDF), respectively. According to the spatial distribution characteristics of the distributed sources, APDF or ASDF can be expressed as a variety of distribution functions, such as Gaussian, uniform and asymmetric distributions [5]. Sources can be one-dimensional (1D) and two-dimensional (2D), depending on the spatial dimension of the sources. Whatever distribution function a source has, the APDF or ASDF of a 1D distributed source is described by two parameters: nominal angles, which can be called direction of arrival (DOA), and angular spreads, which reflect the spatial extension of a source. The APDF or ASDF of a 2D distributed source is depicted by four parameters including the nominal azimuth and nominal elevation, which can be collectively called DOA, and the elevation spread and azimuth spread, which can be called angular spreads reflecting the 2D spatial extension of a source. Compared with the 1D source, which has more parameters, the complexity of estimation for 2D sources significantly increases. In this paper, we are concerned with 2D ID sources.

Regarding point sources, the DOA estimation has recently made some important achievements in the field of mixed signal and multiple input multiple output (MIMO) systems, as well as sparse arrays. Under a uniform rectangular array, the 2D DOA estimation of circular and non-circular mixed signals is proposed in [6]. The authors of [7] have proposed an adaptive beamforming algorithm based on the minimum variance distortionless response principle. With coprime arrays, the authors of [8] have presented a virtual array interpolation estimator; the authors of [9] have proposed a sparse reconstruction using a proposed sliding window scheme for optimization; the authors of [10] have proposed an ESPRIT framework estimator; the authors of [11] have proposed an estimator utilizing Toeplitz matrix functions to resolve off-grid sources. An estimator was proposed in [12] for 2D DOA, based on the least squares technique in the electromagnetic vector sensor MIMO system. The authors of [13] have proposed a DOA estimator based on off-grid sparse Bayesian learning. In [14], the authors have proposed an estimator where transmitted and received DOA are first estimated via trilinear decomposition and refined by 2D searches. 

Considering CD sources, scholars have presented estimators in [15,16,17,18,19,20,21,22,23,24,25]. For 1D ID distributed sources, scholars have proposed various parameter estimation algorithms, mainly including subspace algorithms such as DSPE [1] and DSPARE [4], covariance matching estimation techniques [26,27,28], maximum likelihood estimation algorithms [29,30,31,32] and beamforming algorithms [33,34,35]. For 2D ID sources, the authors of [36] have proposed a 2D covariance matching estimation technique, which uses four-dimensional nonlinear optimization with a large amount of computation. Scholars have proposed low-complexity estimators for 2D ID sources. The authors of [37] have proposed an estimator for DOAs where nominal elevations were first obtained through rotating invariant relations, after which nominal azimuths were solved by a Capon spectral search, which increased computational complexity. 

In this paper, aiming at the parameters of 2D ID sources, an estimator is proposed under double cross arrays. Based on the rotating invariant relations derived through the first order of the Taylor expansion of array manifold vectors, received vectors of arrays are reconstructed via products of extended signal vectors and generalized manifold matrices. Nominal angles can be obtained through signal subspaces of covariance matrices of received vectors. According to the solved nominal angles, angular spreads can be searched via the 2D Capon principle. The main contributions of this article are listed below:

- Generally, the parameters of ID sources constitute an approximate conclusion under the condition of smaller sensor spacing. We propose a double cross array that satisfies both the small space between sensors and the aperture equalization in the elevation and azimuth dimensions.
- Based on the reconstructed received signal vectors and deduced rotating invariant relations, we propose an approach to a solution for nominal angles according to the ESPRIT framework, and we propose an angle matching as well as angular spreads solutions using the Capon estimation.
- The proposed method has an advantage in the DOA estimation with respect to the computational cost, shows advantages in terms of estimation accuracy with a similar number of sensors, and shows robustness in the case of a model mismatch.

The structure of the paper is as follows. Section 2 elaborates the source model and the received vectors of proposed arrays. In Section 3, based on rotating invariant relations and the reconstructed received signal vectors, approaches to a solution for DOA and angular spreads are detailed. Section 4 illustrates the simulation results, which are discussed. Section 5 draws the conclusions.

Notations: Scalar variables are denoted by italic letters, and vectors and matrices are denoted by bold letters. (•)^−1^, (•)*, (•)*^T^* and (•)*^H^* means inverse, complex conjugate, transpose and Hermitian transposition of a matrix. *E*[•], (./) and (•)+ denote expectation, element-wise division and pseudo-inverse operations. [•]*_k_* is the *k*th element of a vector. *angle*(•) is a phase of a complex number.

## 2. 2D ID Source Model and Double Cross Arrays

**Figure 1:**
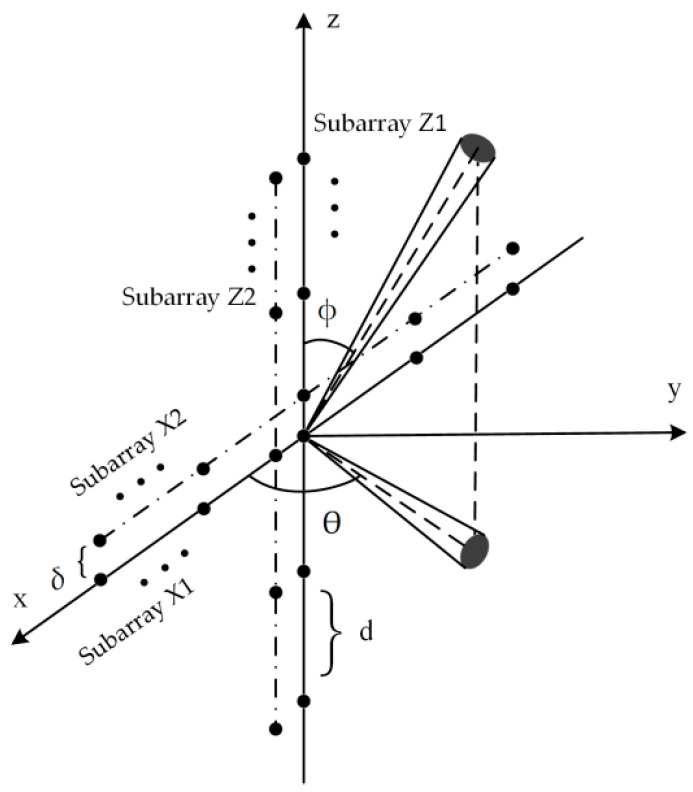
Double cross arrays structure.

The proposed double cross arrays structure is shown in Figure 1. The array is composed of two pairs of parallel linear arrays X1 and X2, and Z1 and Z2. All subarrays are located on the *xoz* plane. Subarrays X1 and Z1 are located on the *x* axis and *z* axis, respectively. Subarrays X1 and Z1 share the sensor located on the origin. Both subarrays X1 and X2 have *M* sensors on both sides of the *z* axis, while both subarrays Z1 and Z2 have *M* sensors on both sides of the *x* axis. All linear arrays have 2*M* + 1 sensors with a spacing of *d* meters. The intervals between each pair of parallel linear arrays are all *δ*.

Assuming that *k* narrow-band 2D ID sources in the far field with nominal angles (*θ_i_*, *φ_i_*) (*i* = 1, 2, …, *k*) are impinging on the array. *θ_i_* and *φ_i_* denote the nominal elevation and nominal azimuth of the *i*th source, respectively. *θ_i_* ∈ [0, π], *φ_i_* ∈ [0, π]. Then, the received vectors of the arrays can be written as:

(1) $$\left\{ \begin{array}{l} x_{m}(t)=\sum_{i=1}^{k}\iint \alpha_{m}(\theta ,\varphi )s_{i}(\theta ,\varphi ,t)d\theta d\varphi +n_{xm}(t)\\ z_{m}(t)=\sum_{i=1}^{k}\iint \beta_{m}(\theta ,\varphi )s_{i}(\theta ,\varphi ,t)d\theta d\varphi +n_{zm}(t) \end{array}\right.,$$

where *m* = 1 or 2. **x**_1_(*t*) and **x**_2_(*t*) represent the received vectors of arrays X1 and X2, respectively; **z**_1_(*t*) and **z**_2_(*t*) are the received vectors of arrays Z1 and Z2. **n***_xm_*(*t*) and **n***_zm_*(*t*) are additive white Gaussian noises which are unrelated to the signals. The noise power is $\sigma_{n}^{2}$. **α**_1_ (*θ*, *φ*) and **α**_2_ (*θ*, *φ*) represent array manifold vectors of the point source with respect to X1 and X2. **β**_1_ (*θ*, *φ*) and **β**_2_ (*θ*, *φ*) denote array manifold vectors of the point source with respect to Z1 and Z2.

(2) $$\left\{ \begin{array}{l} \alpha_{1}(\theta ,\varphi )={\left[e^{j2\pi d(1-M)\cos\theta \sin\varphi /\lambda },\cdots 1,\cdots ,e^{j2\pi (M-1)d\cos\theta \sin\varphi /\lambda }\right]}^{T}\\ \alpha_{2}(\theta ,\varphi )=\alpha_{1}(\theta ,\varphi )e^{j2\pi \delta \cos\varphi /\lambda } \end{array}\right.,$$

(3) $$\left\{ \begin{array}{l} \beta_{1}(\theta ,\varphi )={\left[e^{j2\pi d(1-M)\cos\varphi /\lambda },\cdots ,1,\cdots ,e^{j2\pi (M-1)d\cos\varphi /\lambda }\right]}^{T}\\ \beta_{2}(\theta ,\varphi )=\beta_{1}(\theta ,\varphi )e^{j2\pi \delta \cos\theta \sin\varphi /\lambda } \end{array}\right..$$

*s_i_*(*θ*,*φ*,*t*) is the complex random angular signal density of the *i*th distributed source, representing the scatterer intensity of the source from the direction (*θ*, *φ*) at the time *t*. Unlike a point source, the signal of a distributed source exists not only in a single direction (*θ_i_*, *φ_i_*) but in a spatial distribution around (*θ_i_*, *φ_i_*). An ID source means that different scatterers from one target generate uncorrelated signals. Therefore, one direction from *s_i_*(*θ*,*φ*,*t*) is uncorrelated with other directions from *s_i_*(*θ*,*φ*,*t*), which means that the following relationship exists:

(4) $$E[s_{i}(\theta ,\varphi ,t)s_{i}^{*}(\theta^{\prime },\varphi^{\prime },t)]=\sigma_{i}^{2}f(\theta ,\varphi ;u_{i})\delta (\theta -\theta^{\prime })\delta (\varphi -\varphi^{\prime }),$$

where $\sigma_{i}^{2}$ is the power of the *i*th source, *δ*(•) is the Kronecker delta function and $p(\theta ,\varphi ;u_{i})$ is the normalized angular power density function (APDF). APDF have a parameter set **u***_i_*= [$\theta_{i}$, $\phi_{i}$, $\sigma_{\theta i}$, $\sigma_{\phi i}$] denoting the nominal azimuth, nominal elevation, azimuth spread and elevation spread, respectively. $p(\theta ,\varphi ;u_{i})$ satisfies the following relationship:

(5) $$\iint p(\theta ,\varphi ;u_{i})d\theta d\varphi =1.$$

## 3. Proposed Method

This part is composed of five sections. First, generalized manifold vectors and the rotating invariant relations of generalized manifold vectors are derived. Next, the received signal vectors can be reconstructed as products of generalized signal vectors and generalized manifold vectors. Based on the reconstructed form of the received signal vectors and rotating invariant relations, nominal angles can be resolved separately according to an ESPRIT-like framework. Then, in order to pair the nominal angles, an angle matching approach is proposed under the Capon principle. Afterwards, angular spreads can be obtained by two-dimensional spectral searching using the Capon estimation. Last, the computational procedure is introduced, and the complexity of the proposed approach in comparison with several existing methods is analyzed.

### 3.1. Rotating Invariant Relations

The array manifold vectors of arrays X1 and X2 are expanded with a first-order Taylor series at the point (*θ_i_*, *φ_i_*) as follows:

(6) $$\alpha_{1}(\theta ,\varphi )\approx \alpha_{1}(\theta_{i},\varphi_{i})+[\alpha_{1}{\left(\theta_{i},\varphi_{i}\right]}_{\theta }^{\prime }(\theta -\theta_{i})+[\alpha_{1}{\left(\theta_{i},\varphi_{i}\right]}_{\varphi }^{\prime }(\varphi -\varphi_{i}),$$

(7) $$\alpha_{2}(\theta ,\varphi )\approx \alpha_{2}(\theta_{i},\varphi_{i})+[\alpha_{2}{\left(\theta_{i},\varphi_{i}\right]}_{\theta }^{\prime }(\theta -\theta_{i})+[\alpha_{2}{\left(\theta_{i},\varphi_{i}\right]}_{\varphi }^{\prime }(\varphi -\varphi_{i}),$$

where ${\left[•\right]}_{\theta }^{\prime }$ and ${\left[•\right]}_{\varphi }^{\prime }$ represent the first partial derivative of the function with respect to $\theta_{i}$ and $\varphi_{i}$, respectively. Thus, we have the following relationships:

(8) $$\left\{ \begin{array}{l} \alpha_{2}(\theta_{i},\varphi_{i})=\alpha_{1}(\theta_{i},\varphi_{i})e^{j2\pi \delta \cos\varphi_{i}/\lambda }\\ {\left[\alpha_{2}(\theta_{i},\varphi_{i}\right]}_{\theta }^{\prime }={\left[\alpha_{1}(\theta_{i},\varphi_{i})\right]}_{\theta }^{\prime }e^{j2\pi \delta \cos\varphi_{i}/\lambda }\\ {}[\alpha_{2}{\left(\theta_{i},\varphi_{i}\right]}_{\varphi }^{\prime }={\left[\alpha_{1}(\theta_{i},\varphi_{i})\right]}_{\varphi }^{\prime }e^{j2\pi \delta \cos\varphi_{i}/\lambda }-(j2\pi \delta \sin\varphi_{i}/\lambda )\alpha_{1}(\theta_{i},\varphi_{i})e^{j2\pi \delta \cos\varphi_{i}/\lambda } \end{array}\right..$$

If δ/λ≪1, the second item on the right side of $[\alpha_{2}{\left(\theta_{i},\varphi_{i}\right]}_{\varphi }^{\prime }$ is negligible, and thus the following approximation relationship exists:

(9) $$[\alpha_{2}{\left(\theta_{i},\varphi_{i}\right]}_{\varphi }^{\prime }\approx {\left[a_{1}(\theta_{i},\varphi_{i})\right]}_{\varphi }^{\prime }e^{j2\pi \delta \cos\varphi_{i}/\lambda }.$$

Define the extended signal vectors $\bar{s}=[{\bar{s}}_{1},{\bar{s}}_{2},{\bar{s}}_{3}]$*^H^* and $\tilde{s}=[{\bar{s}}_{1},{\bar{s}}_{3}]$*^H^*. ${\bar{s}}_{1},{\bar{s}}_{2}$ and ${\bar{s}}_{3}$ can be written as follows:

(10) $$\left\{ \begin{array}{l} {\bar{s}}_{1}=[\rho_{10},\rho_{20},\cdots ,\rho_{k0}]\\ {\bar{s}}_{2}=[\rho_{1\theta },\rho_{2\theta },\cdots ,\rho_{k\theta }]\\ {\bar{s}}_{3}=[\rho_{1\varphi },\rho_{2\varphi },\cdots ,\rho_{k\varphi }] \end{array}\right.,$$

where $\rho_{i0}$, $\rho_{i\theta }$ and $\rho_{i\varphi }$ can be expressed as follows:

(11) $$\left\{ \begin{array}{l} \rho_{i0}=\iint s_{i}(\theta ,\varphi ,t)d\theta d\varphi \\ \rho_{i\theta }=\iint (\theta -\theta_{i})s_{i}(\theta ,\varphi ,t)d\theta d\varphi \\ \rho_{i\varphi }=\iint (\varphi -\varphi_{i})s_{i}(\theta ,\varphi ,t)d\theta d\varphi \end{array}\right..$$

$\bar{s}$ is a 3*k* × 1 dimensional vector, and $\tilde{s}$ is a 2*k* × 1 dimensional vector. It can be proven that the following relationship exists [37]:

(12) $$E[\rho_{il}\rho_{in}]=\left\{ \begin{array}{ll} \sigma_{i}^{2} & l=n=0\\ \sigma_{i}^{2}M_{\theta i} & l=n=\theta \\ \sigma_{i}^{2}M_{\varphi i} & l=n=\varphi \\ 0 & l\neq n \end{array}\right.,$$

where $M_{\theta i}$ and $M_{\varphi i}$ can be expressed as follows:

(13) $$M_{\theta i}=\iint {\left(\theta -\theta_{i}\right)}^{2}p_{i}(\theta ,\varphi ,t)d\theta d\varphi ,$$

(14) $$M_{\varphi i}=\iint {\left(\varphi -\varphi_{i}\right)}^{2}p_{i}(\theta ,\varphi ,t)d\theta d\varphi .$$

Since the sources are unrelated, we have:

(15) $$E[\bar{s}{\bar{s}}^{H}]=diag(\Lambda ,M_{\theta }\Lambda ,M_{\varphi }\Lambda ),$$

(16) $$E[\tilde{s}{\tilde{s}}^{H}]=diag(\Lambda ,M_{\varphi }\Lambda ),$$

where:

(17) $$\left\{ \begin{array}{l} \Lambda =diag(\sigma_{1}^{2},\sigma_{2}^{2},\cdots ,\sigma_{k}^{2})\\ M_{\theta }=diag(M_{\theta 1},M_{\theta 2},\cdots ,M_{\theta k})\\ M_{\varphi }=diag(M_{\varphi 1},M_{\varphi 2},\cdots ,M_{\varphi k}) \end{array}\right..$$

The generalized manifold matrix of the subarray X1 is defined as $\left[A_{11},A_{12},A_{13}\right]$. Blocks of the matrix can be expressed as follows:

(18) $$\left\{ \begin{array}{l} A_{11}=[\alpha_{1}(\theta_{1},\varphi_{1}),\alpha_{1}(\theta_{2},\varphi_{2}),\cdots ,\alpha_{1}(\theta_{k},\varphi_{k})]\\ A_{12}=\left[{\left[\alpha_{1}(\theta_{1},\varphi_{1})\right]}_{\theta }^{\prime },{\left[\alpha_{1}(\theta_{2},\varphi_{2})\right]}_{\theta }^{\prime },\cdots ,{\left[\alpha_{1}(\theta_{k},\varphi_{k})\right]}_{\theta }^{\prime }\right]\\ A_{13}=\left[{\left[\alpha_{1}(\theta_{1},\varphi_{1})\right]}_{\varphi }^{\prime },{\left[\alpha_{1}(\theta_{2},\varphi_{2})\right]}_{\varphi }^{\prime },\cdots ,{\left[\alpha_{1}(\theta_{k},\varphi_{k})\right]}_{\varphi }^{\prime }\right] \end{array}\right..$$

Apparently, $\left[A_{11},A_{12},A_{13}\right]$ is a (2*M* + 1) × 3*k* dimensional generalized manifold matrix. The received vector of the X1 array can be written as:

(19) $$x_{1}(t)=[A_{11},A_{12},A_{13}]\bar{s}+n_{x1}(t).$$

The generalized manifold matrix of the subarray X2 is defined as $\left[A_{21},A_{22},A_{23}\right]$, which is (2*M* + 1) × 3*k* dimensional. Blocks of the matrix can be written as follows:

(20) $$\left\{ \begin{array}{l} A_{21}=[\alpha_{2}(\theta_{1},\varphi_{1}),\alpha_{2}(\theta_{2},\varphi_{2}),\cdots ,\alpha_{2}(\theta_{k},\varphi_{k})]\\ A_{22}=\left[{\left[\alpha_{2}(\theta_{1},\varphi_{1})\right]}_{\theta }^{\prime },{\left[\alpha_{2}(\theta_{2},\varphi_{2})\right]}_{\theta }^{\prime },\cdots ,{\left[\alpha_{2}(\theta_{k},\varphi_{k})\right]}_{\theta }^{\prime }\right]\\ A_{23}=\left[{\left[\alpha_{2}(\theta_{1},\varphi_{1})\right]}_{\varphi }^{\prime },{\left[\alpha_{2}(\theta_{2},\varphi_{2})\right]}_{\varphi }^{\prime },\cdots ,{\left[\alpha_{2}(\theta_{k},\varphi_{k})\right]}_{\varphi }^{\prime }\right] \end{array}\right..$$

The received vector of the X2 array can be written as:

(21) $$x_{2}(t)=[A_{21},A_{22},A_{23}]\bar{s}+n_{x2}(t).$$

According to Equations (8) and (9), the following rotating invariant relation can be obtained:

(22) $$[A_{21},A_{22},A_{23}]\approx [A_{11}\Phi ,A_{12}\Phi ,A_{13}\Phi ],$$

where the rotating operator can be written as:

(23) $$\Phi =diag(e^{j2\pi \delta \cos\varphi_{1}/\lambda },e^{j2\pi \delta \cos\varphi_{2}/\lambda },\cdots ,e^{j2\pi \delta \cos\varphi_{k}/\lambda }).$$

The array manifold vectors of arrays Z1 and Z2 are expanded with the first-order Taylor series at the point (*θ_i_*, *φ_i_*) as follows:

(24) $$\beta_{1}(\theta ,\varphi )\approx \beta_{1}(\theta_{i},\varphi_{i})+{\left[\beta_{1}(\theta_{i},\varphi_{i})\right]}_{\varphi }^{\prime }(\varphi -\varphi_{i}),$$

(25) $$\beta_{2}(\theta ,\varphi )\approx \beta_{2}(\theta_{i},\varphi_{i})+{\left[\beta_{2}(\theta_{i},\varphi_{i})\right]}_{\theta }^{\prime }(\theta -\theta_{i})+{\left[\beta_{2}(\theta_{i},\varphi_{i})\right]}_{\varphi }^{\prime }(\varphi -\varphi_{i}),$$

where:

(26) $${\left[\beta_{2}(\theta_{i},\varphi_{i})\right]}_{\theta }^{\prime }=\beta_{1}(\theta_{i},\varphi_{i})(-j2\pi \delta \sin\theta_{i}\sin\varphi_{i}/\lambda )e^{j2\pi \delta \cos\theta_{i}\sin\varphi_{i}/\lambda },$$

(27) $${\left[\beta_{2}(\theta_{i},\varphi_{i})\right]}_{\varphi }^{\prime }={\left[\beta_{1}(\theta_{i},\varphi_{i})\right]}_{\varphi }^{\prime }e^{j2\pi \delta \cos\theta_{i}\sin\varphi_{i}/\lambda }+(j2\pi \delta \cos\theta_{i}\cos\varphi_{i}/\lambda )e^{j2\pi \delta \cos\theta_{i}\sin\varphi_{i}/\lambda }\beta_{1}(\theta_{i},\varphi_{i}).$$

If δ/λ≪1, the right side of Equation (26) and the second item on the right side of Equation (27) can be ignored, then we have the following relationships:

(28) $$\left\{ \begin{array}{l} \beta_{2}(\theta_{i},\varphi_{i})=\beta_{1}(\theta_{i},\varphi_{i})e^{j2\pi \delta \cos\theta_{i}\sin\varphi_{i}/\lambda }\\ {\left[\beta_{2}(\theta_{i},\varphi_{i})\right]}_{\varphi }^{\prime }\approx {\left[\beta_{1}(\theta_{i},\varphi_{i})\right]}_{\varphi }^{\prime }e^{j2\pi \delta \cos\theta_{i}\sin\varphi_{i}/\lambda } \end{array}\right..$$

Define the generalized manifold matrix of array Z1 as $\left[B_{11},B_{12}\right]$, which is (2*M* + 1) × 2*k* dimensional. Blocks in the matrix can be written as follows:

(29) $$\left\{ \begin{array}{l} B_{11}=[\beta_{1}(\theta_{1},\varphi_{1}),\beta_{1}(\theta_{2},\varphi_{2}),\cdots ,\beta_{1}(\theta_{k},\varphi_{k})]\\ B_{12}=\left[{\left[\beta_{1}(\theta_{1},\varphi_{1})\right]}_{\varphi }^{\prime },{\left[\beta_{1}(\theta_{2},\varphi_{2})\right]}_{\varphi }^{\prime },\cdots ,{\left[\beta_{1}(\theta_{k},\varphi_{k})\right]}_{\varphi }^{\prime }\right] \end{array}\right..$$

Then, the received vector of the Z1 array can be written as:

(30) $$z_{1}(t)=[B_{11},B_{12}]\tilde{s}+n_{z1}(t).$$

Define the generalized manifold matrix of array Z2 as $\left[B_{21},B_{22}\right]$, which is (2*M* + 1) × 2*k* dimensional. Blocks in the matrix can be written as follows:

(31) $$\left\{ \begin{array}{l} B_{21}=[\beta_{2}(\theta_{1},\varphi_{1}),\beta_{2}(\theta_{2},\varphi_{2}),\cdots ,\beta_{2}(\theta_{k},\varphi_{k})]\\ B_{22}=\left[{\left[\beta_{2}(\theta_{1},\varphi_{1})\right]}_{\varphi }^{\prime },{\left[\beta_{2}(\theta_{2},\varphi_{2})\right]}_{\varphi }^{\prime },\cdots ,{\left[\beta_{2}(\theta_{k},\varphi_{k})\right]}_{\varphi }^{\prime }\right] \end{array}\right..$$

The received vector of subarray Z2 can be written as:

(32) $$z_{2}(t)=[B_{21},B_{22}]\tilde{s}+n_{z2}(t).$$

According to (28), the following rotating invariant relation is obtained:

(33) $$[B_{21},B_{22}]\approx [B_{11}\Psi ,B_{12}\Psi ],$$

where the rotating operator can be written as:

(34) $$\Psi =diag(e^{j2\pi \delta \cos\theta_{1}\sin\varphi_{1}/\lambda },e^{j2\pi \delta \cos\theta_{2}\sin\varphi_{2}/\lambda },\cdots ,e^{j2\pi \delta \cos\theta_{k}\sin\varphi_{k}/\lambda }).$$

### 3.2. Estimation of Nominal Angles

Combine the received vectors of subarrays X1 and X2 as follows:

(35) $$x_{12}(t)=\left[\begin{array}{l} x_{1}(t)\\ x_{2}(t) \end{array}\right]=\left[A_{x1},A_{x2},A_{x3}\right]\bar{s}+n_{x}(t),$$

where $\left[A_{x1},A_{x2},A_{x3}\right]$ is the generalized manifold matrix of the combined received vectors **x**_12_(*t*), which can be written as:

(36) $$\left[A_{x1},A_{x2},A_{x3}\right]=\left[\begin{array}{l} A_{11},A_{12},A_{13}\\ A_{21},A_{22},A_{23} \end{array}\right].$$

The combined noise vector of subarrays X1 and X2 can be written as:

(37) $$n_{x}(t)=\left[\begin{array}{l} n_{x1}(t)\\ n_{x2}(t) \end{array}\right].$$

The covariance matrix of the combined receive vectors **x**_12_(*t*) can be written as:

(38) $$R_{x}^{12}=E[x_{12}(t)x_{12}^{H}(t)].$$

The sample covariance matrix ${\hat{R}}_{x}^{12}$ can be substituted for $R_{x}^{12}$ in calculation, having the following expression:

(39) $${\hat{R}}_{x}^{12}=\frac{1}{N}\sum_{t=1}^{N}x_{12}(t)x_{12}^{H}(t).$$

For ID sources, the rank of the covariance matrix is theoretically larger than the source number. Define $E_{x}$ as a subspace formed by the eigenvectors corresponding to the maximum *k* eigenvalues of the covariance matrix $R_{x}^{12}$. As both *M_θi_* and *M_φi_* are numbers far below 1 with the condition of a small angular spread, the subspace $E_{x}$ is the same as the subspace spanned by $A_{x1}$. Then, there exists a *k* × *k* nonsingular matrix **T** that satisfies the following relationship:

(40) $$E_{x}=\left[\begin{array}{l} A_{11}\\ A_{11}\Phi \end{array}\right]T,$$

where Φ is the rotating operator described by Equation (23), **A**_11_ is the first block of the generalized manifold matrix of the subarray X1 described by Equation (18).

Define $E_{x1}$ and $E_{x2}$ as the upper and lower *k*th rows of $E_{x}$, then we have:

(41) $$E_{x1}=A_{11}T.$$

(42) $$E_{x2}=A_{21}T.$$

Thus, the following relationship can be obtained:

(43) $$E_{x2}=E_{x1}T^{-1}\Phi T.$$

Define $\Omega_{x}=T^{-1}\Phi T$, we have:

(44) $$\Omega_{x}=E_{x1}^{+}E_{x2}.$$

Therefore, the nominal elevations of the ID sources can be solved as follows:

(45) $$\varphi_{i}=\arccos\frac{angle(\eta_{i})}{2\pi \delta /\lambda }i=1,2,\cdots ,k,$$

where $\eta_{i}$ is the *i*th eigenvalue of $\Omega_{x}$.

Combine the received vectors of subarrays Z1 and Z2 as follows:

(46) $$z_{12}(t)=\left[\begin{array}{l} z_{1}(t)\\ z_{2}(t) \end{array}\right]=\left[B_{z1},B_{z2}\right]\tilde{s}+n_{z}(t),$$

where the generalized manifold matrix of the combined received vectors **z**_12_(*t*) can be written as:

(47) $$\left[B_{z1},B_{z2}\right]=\left[\begin{array}{l} B_{11},B_{12}\\ B_{21},B_{22} \end{array}\right].$$

The combined noise vector of subarrays Z1 and Z2 can be written as:

(48) $$n_{z}(t)=\left[\begin{array}{l} n_{z1}(t)\\ n_{z2}(t) \end{array}\right].$$

The covariance matrix of the combined received vectors **z**_12_(*t*) can be expressed as:

(49) $$R_{z}^{12}=E[z_{12}(t)z_{12}^{H}(t)].$$

The sample covariance matrix ${\hat{R}}_{z}^{12}$ can be substituted for $R_{z}^{12}$ in calculation, having the following expression:

(50) $${\hat{R}}_{z}^{12}=\frac{1}{N}\sum_{t=1}^{N}z_{12}(t)z_{12}^{H}(t).$$

As *M_φi_* is a number below 1 under the condition of a small angular spread, the subspace $E_{z}$ constructed by eigenvectors corresponding to the maximum *k* eigenvalues of the covariance matrix $R_{z}^{12}$ is the same as the subspace spanned by $B_{z1}$. Then, there exists a *k* × *k* nonsingular matrix **Q** that satisfies the following relationship:

(51) $$E_{z}=\left[\begin{array}{l} B_{11}\\ B_{11}\Psi \end{array}\right]Q,$$

where Ψ is the rotating operator described by Equation (34), **B**_11_ is the first block of the generalized manifold matrix of the subarray Z1 described by Equation (29).

Define $E_{z1}$ and $E_{z2}$ as the upper and lower *k*th rows of $E_{z}$:

(52) $$E_{z1}=B_{11}Q,$$

(53) $$E_{z2}=B_{21}Q.$$

Thus, we can obtain:

(54) $$E_{z2}=E_{z1}Q^{-1}\Psi Q.$$

Define $\Omega_{z}=Q^{-1}\Psi Q$, we have:

(55) $$\Omega_{z}=E_{z1}^{+}E_{z2}.$$

Then, the nominal azimuths of the ID sources can be obtained as follows:

(56) $$\theta_{i}=\arccos\frac{angle(\mu_{i})}{2\pi \delta /\lambda \sin\varphi_{i}}i=1,2,\cdots ,k,$$

where $\mu_{i}$ is the *i*th eigenvalue of $\Omega_{z}$.

### 3.3. Angle Matching

Considering the combined generalized manifold matrix $\left[A_{x1},A_{x2},A_{x3}\right]$ of parallel subarrays X1 and X2, the Capon estimation criterion has the following expression:

(57) $$\begin{array}{ll} \min & w^{H}R_{x}^{12}w\\ s.t. & w^{H}\left[A_{x1},A_{x2},A_{x3}\right]=1 \end{array}$$

where $R_{x}^{12}$ is the covariance matrix of the combined received vectors **x**_12_(*t*) described by Equation (38), and **w** is an unknown coefficient vector.

The above equation can be solved by the Lagrange function. The Capon cost function of subarrays X1 and X2 can be obtained as:

(58) $$L(\theta ,\varphi )=\frac{1}{{\left\|{\left[A_{x1},A_{x2},A_{x3}\right]}^{H}{\left({\hat{R}}_{x}^{12}\right)}^{-1}\left[A_{x1},A_{x2},A_{x3}\right]\right\|}_{F}}.$$

The steps for angle matching can be summarized as follows:

1. Choose ${\hat{\varphi }}_{i}=\varphi_{i}$ as the estimated nominal elevation from the set $\left\{\varphi_{1},\varphi_{2},\cdots ,\varphi_{k}\right\}$, traverse the set $\left\{angle(\mu_{1}),angle(\mu_{2}),\cdots ,angle(\mu_{k})\right\}$ and solve the paired nominal angles $\left(\theta_{j},{\hat{\varphi }}_{i}\right)$ (j=1,2,⋯,k) according to Equation (56).
2. Substitute $\left(\theta_{j},{\hat{\varphi }}_{i}\right)$ (j=1,2,⋯,k) into (58) to select the pair that makes the maximum value of L(θ,φ) and denote the pair as $\left({\hat{\theta }}_{j},{\hat{\varphi }}_{i}\right)$.
3. Remove ${\hat{\varphi }}_{i}$ from the set $\left\{\varphi_{1},\varphi_{2},\cdots ,\varphi_{k}\right\}$ and remove $2\pi \delta /\lambda \cos{\hat{\theta }}_{i}\sin{\hat{\varphi }}_{i}$ from the set $\{angle(\mu_{1}),angle(\mu_{2}),\cdots ,$ $angle(\mu_{k})\}$.
4. Repeat steps 1–3.

All nominal angles can be paired by (*k* + 2)(*k* − 1)/2 calculations.

### 3.4. Estimation of Angular Spread

Considering subarray X1, the covariance matrix of the received vector has the following expression:

(59) $$R(\theta ,\varphi ;u_{i})=\iint p(\theta ,\varphi ;u_{i})\alpha_{1}(\theta ,\varphi )\alpha_{1}^{H}(\theta ,\varphi )d\theta d\varphi .$$

For Gaussian 2D ID sources, APDF has the following expression:

(60) $$p(\theta ,\varphi ;u_{i})=\frac{1}{2\pi \sigma_{\theta i}\sigma_{\varphi i}}\exp\left\{-0.5\left[{\left(\frac{\theta -\theta_{i}}{\sigma_{\theta i}}\right)}^{2}+{\left(\frac{\varphi -\varphi_{i}}{\sigma_{\varphi i}}\right)}^{2}\right]\right\}.$$

Assuming d/λ=0.5, and within the premise of small angular spreads, the elements of $R(\theta ,\varphi ;u_{i})$ can be expressed as follows:

(61) $${\left[R(\theta ,\varphi ;u_{i})\right]}_{lh}=e^{j\pi (l-h)\cos\theta_{i}\sin\varphi_{i}}e^{-0.5\left\{{\left[\pi \sigma_{\theta i}(l-h)\sin\theta_{k}\sin\varphi_{i}\right]}^{2}+{\left[\pi \sigma_{\varphi i}(l-h)\cos\theta_{i}\cos\varphi_{i}\right]}^{2}\right\}},$$

where [•]*_lh_* represents the element of the *l*th row and *h*th column of a matrix.

For uniform 2D ID sources, APDF has the following expression:

(62) $$p(\theta ,\varphi ;u_{i})=\left\{ \begin{array}{ll} \frac{1}{4\sigma_{\theta i}\sigma_{\varphi i}} & \left|\theta -\theta_{i}\right|\leq \sigma_{\theta i}\;\mathrm{and}\;\left|\varphi -\varphi_{i}\right|\leq \sigma_{\varphi i}\\ 0 & \left|\theta -\theta_{i}\right|\geq \sigma_{\theta i}\mathrm{or}\left|\varphi -\varphi_{i}\right|\geq \sigma_{\varphi i} \end{array}\right..$$

$R(\theta ,\varphi ;u_{i})$ has the following expression:

(63) $${\left[R(\theta ,\varphi ;u_{i})\right]}_{lh}=e^{j\pi (l-h)\cos\theta_{i}\sin\varphi_{i}}\frac{\sin[\pi \sigma_{\varphi i}(l-h)\cos\theta_{i}\cos\varphi_{i}]\cdot \sin[\pi \sigma_{\theta i}(l-h)\sin\theta_{i}\sin\varphi_{i}]}{\pi \sigma_{\varphi i}(l-h)\cos\theta_{i}\cos\varphi_{i}\cdot \pi \sigma_{\theta i}(l-h)\sin\theta_{i}\sin\varphi_{i}}.$$

Azimuth spreads as well as elevation spreads can be obtained by two-dimensional spectral searching using the Capon estimation:

(64) $$({\hat{\sigma }}_{\theta },{\hat{\sigma }}_{\varphi })=\arg\max\frac{1}{\sigma_{\max}\{{\left({\hat{R}}_{x}^{1}\right)}^{-1}R(\theta ,\varphi ;u_{i})\}},$$

where $\sigma_{\max}\{•\}$ represents the maximum eigenvalue of the matrix. ${\hat{R}}_{x}^{1}$ is the sample covariance matrix of arrays X1, having the following expression:

(65) $${\hat{R}}_{x}^{1}=\frac{1}{N}\sum_{t=1}^{N}x_{1}(t)x_{1}^{H}(t).$$

### 3.5. Algorithm Procedure and Complexity Analysis

According to the above analysis, the algorithm steps are summarized as follows:

1. Calculate the sample covariance matrix ${\hat{R}}_{x}^{12}$ and ${\hat{R}}_{z}^{12}$.
2. Obtain $E_{x}$ and $E_{z}$ by decomposition of ${\hat{R}}_{x}^{12}$ and ${\hat{R}}_{z}^{12}$. Calculate $\Omega_{x}$ and $\Omega_{z}$ according to (44) and (55).
3. Conduct the decomposition of $\Omega_{x}$ and $\Omega_{z}$ to obtain the eigenvalues $\eta_{i}$ and $\mu_{i}$, and then calculate the nominal elevations $\varphi_{i}$ and nominal azimuths $\theta_{i}$ from (45) and (56).
4. Determine the nominal angles $\left({\hat{\theta }}_{i},{\hat{\varphi }}_{i}\right)$ (j=1,2,⋯,k) according to the angle matching method.
5. Substitute $\left({\hat{\theta }}_{i},{\hat{\varphi }}_{i}\right)$ (j=1,2,⋯,k) into $R(\theta ,\varphi ;u_{i})$ according to Equation (61) or (63). Solve the angular spreads parameters ${\hat{\sigma }}_{\theta i}$ and ${\hat{\sigma }}_{\varphi i}$ from (64).

**Figure 2:**
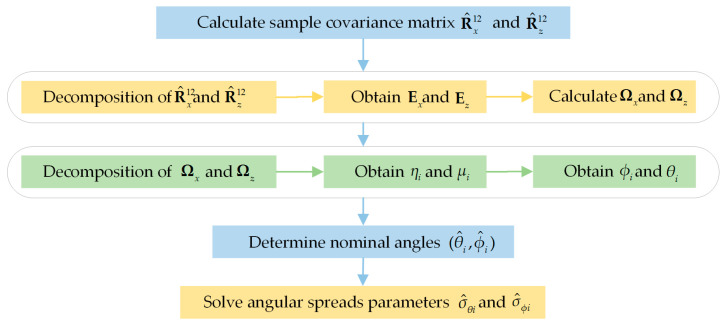
Flowchart of the proposed algorithm.

The flow of the algorithm is shown in Figure 2.

The complexity of the algorithm proposed in this paper with respect to nominal angles contains four parts. First, calculating the covariance matrix ${\hat{R}}_{x}^{12}$ and ${\hat{R}}_{z}^{12}$ is O(*NK*^2^), where *N* is the snapshots number and *K* is the total sensor number. The second part is the eigendecomposition of ${\hat{R}}_{x}^{12}$ and ${\hat{R}}_{z}^{12}$, which is O(*K*^3^). The third is the eigendecomposition of $\Omega_{x}$ and $\Omega_{z}$, which is O(*k*^3^). The fourth is angle matching, which is *O*(*k^2^*). The computational cost of reference [36] mainly contains calculating sample covariance matrices O(*NK*^2^) and the alternating projection technique, which require four-dimensional optimizations: O(*K*^4^). The complexity of the estimation method of double parallel arrays proposed in reference [37] includes three parts. First, calculating the covariance matrix is O(*NK*^2^). The second part is the eigendecomposition of the covariance matrix, which is O(*K*^3^). Third, the 1D search is O(*kL_φ_K*^2^), where *L_φ_* constitutes the search points of the nominal azimuth. The computational complexity of the angular spread parameters is mainly divided into two parts: the inversion of the covariance matrix ${\hat{R}}_{x}^{1}$, which is O[(*K/4*)^3^], and the 2D search to obtain the angular spread parameters, which is O[(*K/4*)^3^*kLσ_θ_Lσ_φ_*], where *Lσ_θ_* and *Lσ_φ_* are the search points of the elevation spread and azimuth spread, respectively. As reference [37], which deals with the problem of DOA estimation, does not involve the estimation of angular spreads, Table 1 shows the main computational costs of three methods for DOA estimation. From Table 1, we can clearly obtain that the computational cost for the DOA estimation of the proposed algorithm is lower than for the algorithms in refs. [36,37]. 

It should be noted that reference [36] also deals with the estimation of angular spreads, where the computational cost for angular spreads is involved in a four-dimensional optimization, O(*K*^4^). From a previous analysis, the computational cost of the proposed method for angular spreads is O[(*K/4*)^3^*kLσ_θ_Lσ_φ_*]. Generally speaking, angular spreads are within a few degrees for 2D ID sources. Supposing both *Lσ_θ_* and *Lσ_φ_* are set with 20 search points, which means that the search range is 2° with a step size 0.1°, when *K* > 6.25*k* the computational cost for angular spreads in reference [36] is larger than the proposed one. To summarize, the proposed DOA estimation has a significant advantage in terms of computational cost, whereas the angular spreads are not obvious.

## 4. Results and Discussions

The arrays structure is set with each linear array containing five sensors, which means that the total sensor number of the double cross arrays is 19. The array element spacing is set as d=λ/2 and the parallel array interval is δ=λ/10. The root mean square error (RMSE) of the DOA is defined as *RMSE_a_* with following expression:

(66) $$RMSE_{a}=\sqrt{\frac{1}{Mc}\sum_{\varsigma }^{Mc}{\left({\hat{\theta }}^{\varsigma }-\theta \right)}^{2}+\frac{1}{Mc}\sum_{\varsigma }^{Mc}{\left({\hat{\varphi }}^{\varsigma }-\varphi \right)}^{2}}.$$

*MC* represents the Monte Carlo simulation times, while the superscript ^ and variable ς of ${\hat{\theta }}^{\varsigma }$ and ${\hat{\varphi }}^{\varsigma }$ represent the estimated value in the ςth Monte Carlo simulation. RMSE of the angular spread is defined as *RMSE_s_* with following expression:

(67) $$RMSE_{s}=\sqrt{\frac{1}{Mc}\sum_{\varsigma }^{Mc}{\left({\hat{\sigma }}_{\theta }^{\varsigma }-\sigma_{\theta }\right)}^{2}+\frac{1}{Mc}\sum_{\varsigma }^{Mc}{\left({\hat{\sigma }}_{\varphi }^{\varsigma }-\sigma_{\varphi }\right)}^{2}},$$

where ${\hat{\sigma }}_{\theta }^{\varsigma }$ and ${\hat{\sigma }}_{\varphi }^{\varsigma }$ represent the estimated azimuth spread and elevation spread in the ςth Monte Carlo simulation. *RMSE_a_* denotes the estimation errors of DOA, while *RMSE_s_* denotes the estimation errors of the angular spreads. The signal-to-noise ratio (SNR) is defined as 10log(1/$\sigma_{n}^{2}$), where the noise is assumed to be the Gaussian white zero mean with a variance of $\sigma_{n}^{2}$.

**Figure 3:**
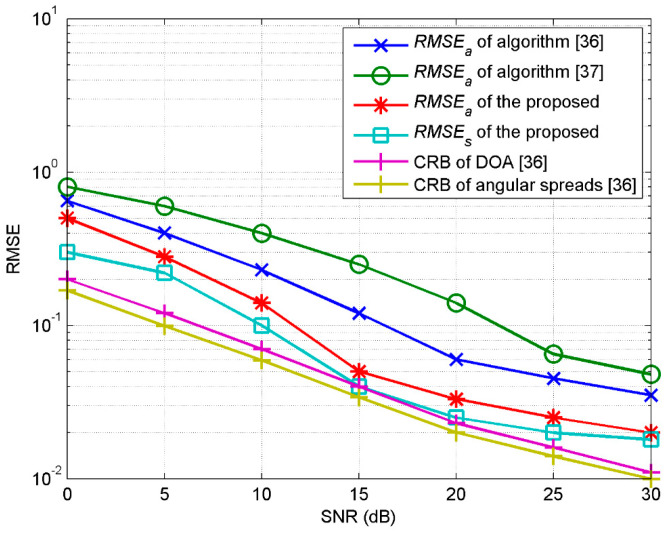
RMSE estimated with the change of SNR.

**Figure 4:**
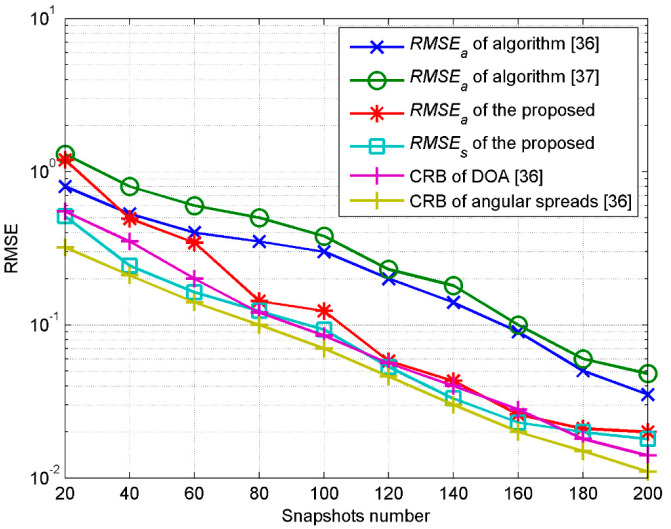
RMSE estimated with the snapshots number.

In the first experiment, we investigate the influence of SNR and the snapshots number on the estimation. A 2D ID Gaussian source with parameters of [30°, 45°, 2°, 3°] is set to be estimated. The experiment is completed by 100 independent Monte Carlo simulations. Figure 3 shows the estimated *RMSE_a_* and *RMSE_s_* with SNR from 0 to 30 db when the snapshots number is equal to 200. Figure 4 shows the estimated *RMSE_a_* and *RMSE_s_* when changing the snapshots number when SNR is equal to 20 db. Figure 3 and Figure 4 also show the estimation results by the method used in refs. [36,37], using 20 sensors based on a uniform rectangular array (URA) and double parallel linear arrays (DPLA), respectively, as well as the Cramer–Rao bound (CRB) [36]. It can be seen that with the improvement of SNR or the snapshots number, the estimation accuracy of the proposed algorithm in this paper is better than that of the algorithm proposed in refs. [36,37] under the same experimental conditions. The method used in reference [37] unitizes double parallel arrays X1and X2 with sensor spacing in the elevation dimension of less than one tenth of the impinging signal wavelength and sensor spacing in the azimuth dimension of less than half of the impinging signal wavelength; consequently, the physical aperture of the array in the elevation dimension is far less than that of the azimuth dimension. Therefore, during the iterative estimation, the estimation of the nominal elevation will produce larger errors, which will also be transmitted to the solution of the nominal azimuth. Though the URA of the method used in reference [36] has a structural balance in the elevation and azimuth dimensions, the spacing of sensors of the double cross array proposed in both the elevation and azimuth dimensions is less than half of the impinging wavelength when the number of elements is constant; the aperture of the array proposed in this paper is larger than the URA in the two dimensions, exhibiting a higher estimation accuracy. To summarize, the advantage of the proposed double cross arrays is that they satisfy both the premise of a small interval of parallel linear arrays and the aperture equalization in the elevation and azimuth dimensions when compared with URA and DPLA.

**Figure 5:**
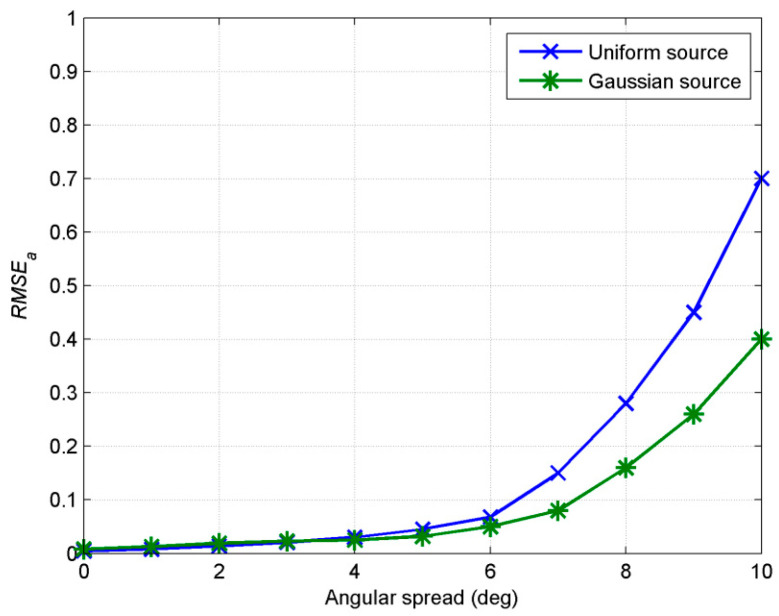
RMSE_a_ estimated with angular spreads.

In the second experiment, we examine the influence of the angular spread on estimation. For a 2D ID source with nominal angles (60°, 35°), two kinds of APDF (Gaussian and uniform) are considered, respectively. The azimuth spreads and elevation spreads are supposed to be equivalent in both kinds of sources. The estimated *RMSE_a_* with an angular spread from 0° to 10° is investigated. The experiment is completed by 100 independent Monte Carlo simulations with the SNR at 20 db and snapshots number at 200. Figure 5 shows that the estimation accuracy decreases with an increase of the angular spreads. For the Gaussian source, when the angular spreads are 5°, *RMSE_a_* is 0.03. As the angular spreads reach 10°, *RMSE_a_* is 0.4. For the uniform source, *RMSE_a_* is 0.04 and 0.7 when the angular spreads are 5° and 10°, respectively. These experimental results show that the estimation performance decreases with the increase of the angular spreads, but that it is still satisfactorily within 10°. The proposed algorithm is robust in the case of small angular spreads.

As can be seen from Figure 5, the DOA estimation accuracy is highest when the angular spreads are 0°. As a matter of fact, whether it is a Gaussian or uniform distributed source or any other distributed sources, when the angular spreads are 0°, the parameters describing the ID source only include the DOA parameters, which means that the distributed source can be equal to the point source. This experiment also shows that the method proposed in this paper is not only suitable for the DOA estimation of the ID source but also suitable for the DOA estimation of the point source. In the case of a mismatch between the point source and ID source model, it also has good robustness.

**Figure 6:**
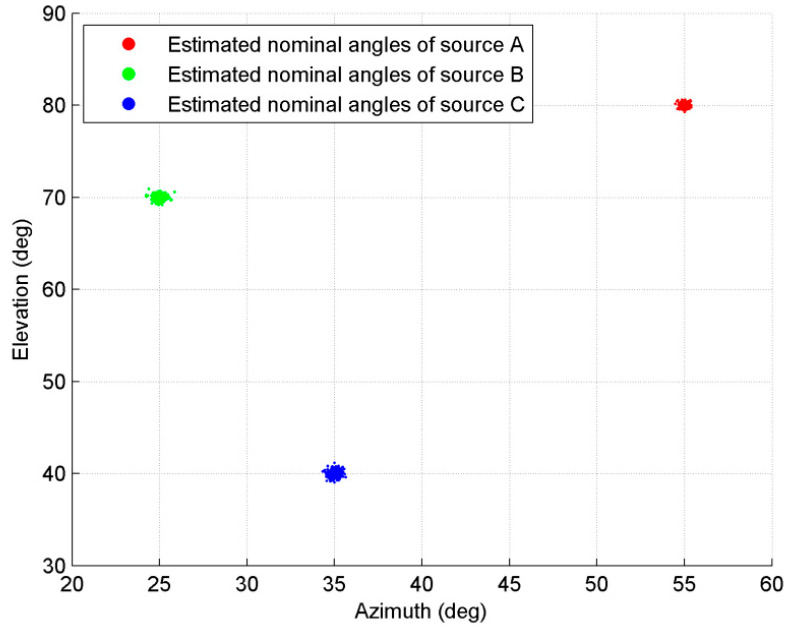
Estimated DOAs of three sources.

**Figure 7:**
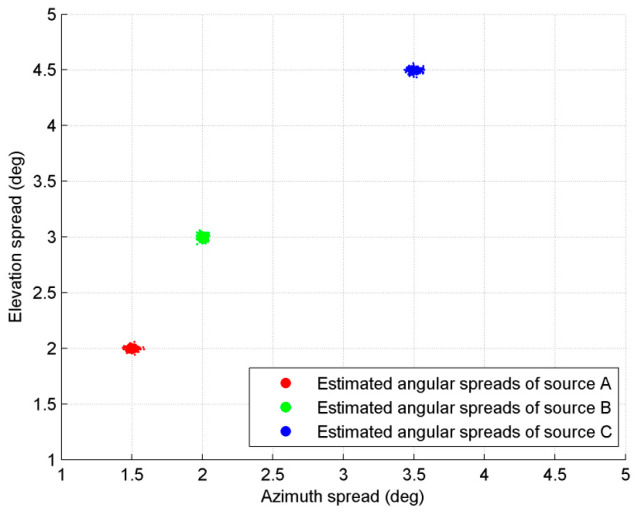
Estimated angular spreads of three distributed sources.

In the third experiment, we investigate the ability of the algorithm to estimate multiple distributed sources. Three ID sources, A, B and C, are set to be estimated. A and B are ID sources with a Gaussian APDF, C is an ID source with a uniform APDF. The three sources have the following parameters: [55°, 80°, 1.5°, 2°], [25°, 70°, 2°, 3°] and [35°, 40°, 3.5°, 4.5°]. SNR is set at 20 db, and the snapshots number is 200. The experiment is completed by 100 independent Monte Carlo simulations. Figure 6 shows the 100 results of the estimated DOAs of the three sources. Figure 5 illustrates that the proposed method can estimate nominal angles of multiple 2D ID source simultaneously without prior knowledge of APDF. Figure 7 shows the 100 results of the estimated angular spreads of the three sources, respectively. From the derivation process in Parts 3.2 and 3.3, as well as from the experimental results, we can see that the method proposed in this paper does not require knowledge of the specific form of APDF in the DOA estimation. As the angular spreads are parameters of the APDF function, it is impossible to discuss the angular spreads parameter without the APDF function. The method proposed in this paper does not need to know the APDF in the DOA estimation, but solving the angular spreads parameters requires prior information on the APDF form first, followed by searching and optimizing. 

It should be noted that the rotating invariant relations in this paper are based on the assumption that the interval of the parallel linear array satisfies a small spacing. In practice, if the spacing of sensors is small, on the one hand, when taking into account the characteristics of the sensors, the mutual coupling effect between the sensors cannot be ignored. On the other hand, the installation error of sensors will also affect the estimation effect. Therefore, this is difficult to apply in some high frequency signal fields such as mobile communication, as the frequency of mobile communication rises from 800 MHz to 2.5 GHz. However, in the low-frequency sonic detection of the underwater background, the sonar frequency can be reduced to 100 HZ, and the signal wavelength can reach approximately 14.5 m. Similarly, with respect to a very high frequency (VHF) radar, the general working wavelength is selected to be within 1–3 m. In these cases, the interval of the parallel linear array can actually reach a value that satisfies the hypothesis.

## 5. Conclusions

In this paper, an estimation of the parameters of a 2D ID source is presented based on a double cross array that satisfies both the small interval of parallel linear arrays and the aperture equalization in the elevation and azimuth dimensions. The rotating invariant relations of the generalized manifold vectors with respect to nominal angles are derived under the proposed array. Received vectors are reconstructed as products of generalized manifold matrices and extended signal vectors by taking the first-order Taylor expansion of the array manifold vector. DOAs are calculated on the basis of the covariance matrix of the received vectors according to a proposed ESPRIT-like framework. Angular spreads are obtained by a two-dimensional search. Numerical simulations are conducted to verify the proposed method while considering different experimental conditions, angular spreads and multiple sources. The outcomes show that the proposed method shows a better estimation performance under the same experimental condition and sensor number when compared with existing methods.

## Figures and Tables

**Table 1:** Computational complexity of different methods for DOA estimation.

Method	Sample Covariance Matrix	Eigendecomposition/Alternating Projection Technique	Searching	Total
Proposed	O(*NK*^2^)	O(*K*^3^) + O(*k*^3^)		O(*NK*^2^) + O(*K*^3^) + O(*k*^3^)
Reference [36]	O(*NK*^2^)	O(*K*^4^)		O(*NK*^2^) + O(*K*^4^)
Reference [37]	O(*NK*^2^)	O(*K*^3^)	O(*kL_φ_K*^2^)	O(*NK*^2^) + O(*K*^3^) + O(*kL_φ_K*^2^)
